# Editorial: Identification of effective biomarkers for diagnosis and treatment of chronic kidney disease: integrating bioinformatics and pharmacological approaches

**DOI:** 10.3389/fphar.2025.1606173

**Published:** 2025-05-07

**Authors:** Swayam Prakash Srivastava, Keizo Kanasaki

**Affiliations:** ^1^ Life Sciences Institute, University of Michigan, Ann Arbor, MI, United States; ^2^ Department of Internal Medicine 1, Faculty of Medicine, Shimane University, Izumo, Japan

**Keywords:** chronic kidney disease, network phamacology, drug-drug interaction, molecular docking, SGLT-2 inhibitor, ANGPTL4, AcSDKP, sirt3

Kidney disease affects more than 850 million people globally, and by the year 2040, it is assumed to be the fifth leading cause of mortality. The Global Burden of Disease reports indicate that the prevalence of chronic kidney disease (CKD) increased by 33% worldwide between 1990 and 2017 (Francis et al., 2024). CKD is a remarkable public health concern worldwide and is associated with increased morbidity, mortality, and healthcare costs. The prevalence of CKD is expected to rise given its association with aging, diabetes, and hypertension. The estimated prevalence of CKD is around 10%–14% of the total population, and a great number of people with CKD do not exhibit symptoms until it advances to kidney failure (Kovesdy, 2022). Chronic kidney disease is a complex kidney disease characterized by impaired renal structure and renal function, persistent inflammation, and moderate immune infiltrations. The consequences of CKD include the irreversible and significant loss of nephrons, tubular damage, chronic immune cell filtrations, reduced regeneration capability, renal macro and microvascular injuries, and aberrant metabolic disarrangements and renal fibrosis, finally leading to kidney failure (Panizo et al., 2021). Renal fibrosis is a leading cause of death in CKD and contributes to kidney failure (O'Callaghan-Gordo et al., 2019). Renal fibrosis in CKD is driven by several key signaling pathways, such as inductions of transforming growth factor beta (TGF-β), Wingless/Int-1 (WNT), angiopoietin-like 4 (ANGPTL4), Hedgehog, and NOTCH signaling pathways, dipeptidyl Peptidase-4 (DPP-4)-integrin β1-mediated mechanisms, and mineralocorticoid receptor-related mechanisms. In contrast, protective pathways, such as glucocorticoid receptors, sirtuin 3 (SIRT3), and fibroblast growth factor receptors, suppress the fibrosis process in CKD, (Srivastava et al., 2024; Srivastava and Goodwin, 2023; Srivastava et al., 2021a; Srivastava et al., 2021b; Li et al., 2020a; Srivastava et al., 2020a; Srivastava et al., 2021c; Bhatia and Srivastava, 2023).

Despite the importance of early detection and management of CKD, there is a lack of reliable diagnostic biomarkers and effective therapeutic targets for this condition. This presents a significant clinical challenge to clinicians and researchers. Therefore, there is an urgent need to explore new strategies for the diagnosis and management of CKD. Several therapeutic agents are being evaluated for their efficacy in preclinical models. Sodium-glucose cotransporter 2 (SGLT2) inhibitors have shown significant antifibrotic effects in mouse models by inhibiting the partial epithelial-mesenchymal transition (EMT) and restoring kidney structure and function (Li et al., 2020b). Empagliflozin is a drug belonging to the class of SGLT-2 inhibitors, and it has been shown to induce mitochondrial protein SIRT3 levels and elevate the levels of fatty acid oxidation; these mechanisms are found to be suppressed in injured kidneys (Li et al., 2020b). Concomitantly, empagliflozin significantly suppressed the abnormal glycolysis and related kidney fibrosis in a mouse model of CKD (Li et al., 2020b). This aberrant glycolysis mediated through pyruvate kinase M2 type dimers and hypoxia inducible factor 1a (HIF-1a) is the causative mechanism for the development of CKD (Li et al., 2020b; Srivastava et al., 2018). SIRT3 activation agents, such as honokiol and 5-Aminoimidazole-4-carboxamide ribonucleotide (AICAR), have shown protective effects against CKD (Wei et al., 2023). Linagliptin, a DPP-4 inhibitor, exhibits reno-protective effects beyond diabetes (Srivastava et al., 2020a; Kanasaki et al., 2014; Shi et al., 2015). In addition, linagliptin inhibited partial EMT and endothelial-to-mesenchymal transition (EndMT) by suppressing hypoxia-related proteins and Snail and Twist levels. Rho-associated protein kinase 2 (ROCK2)-TGF-β1-EMT and ROCK2-nuclear factor erythroid 2-related factor 2 (Nrf2) pathways may offer a novel therapeutic strategy for the treatment of CKD (You et al., 2020). However, a large cohort clinical trial is needed to analyze its effects in human subjects. Conventional treatment involves reno-protective agents such as angiotensin converting enzyme inhibitors (ACEis) and angiotensin receptor blockers (ARBs), and both classes of drugs lower the hypertension-related fibrogenesis in the kidney (Nagai et al., 2014; Nitta et al., 2016). AcSDKP has been found to be effective in lowering the late-stage fibrosis in mouse models of CKD by elevating SIRT3 protein levels and improving mitochondrial function in tubules, podocytes, and endothelial cells (Srivastava et al., 2020b; Srivastava et al., 2020c).

**FIGURE 1:**
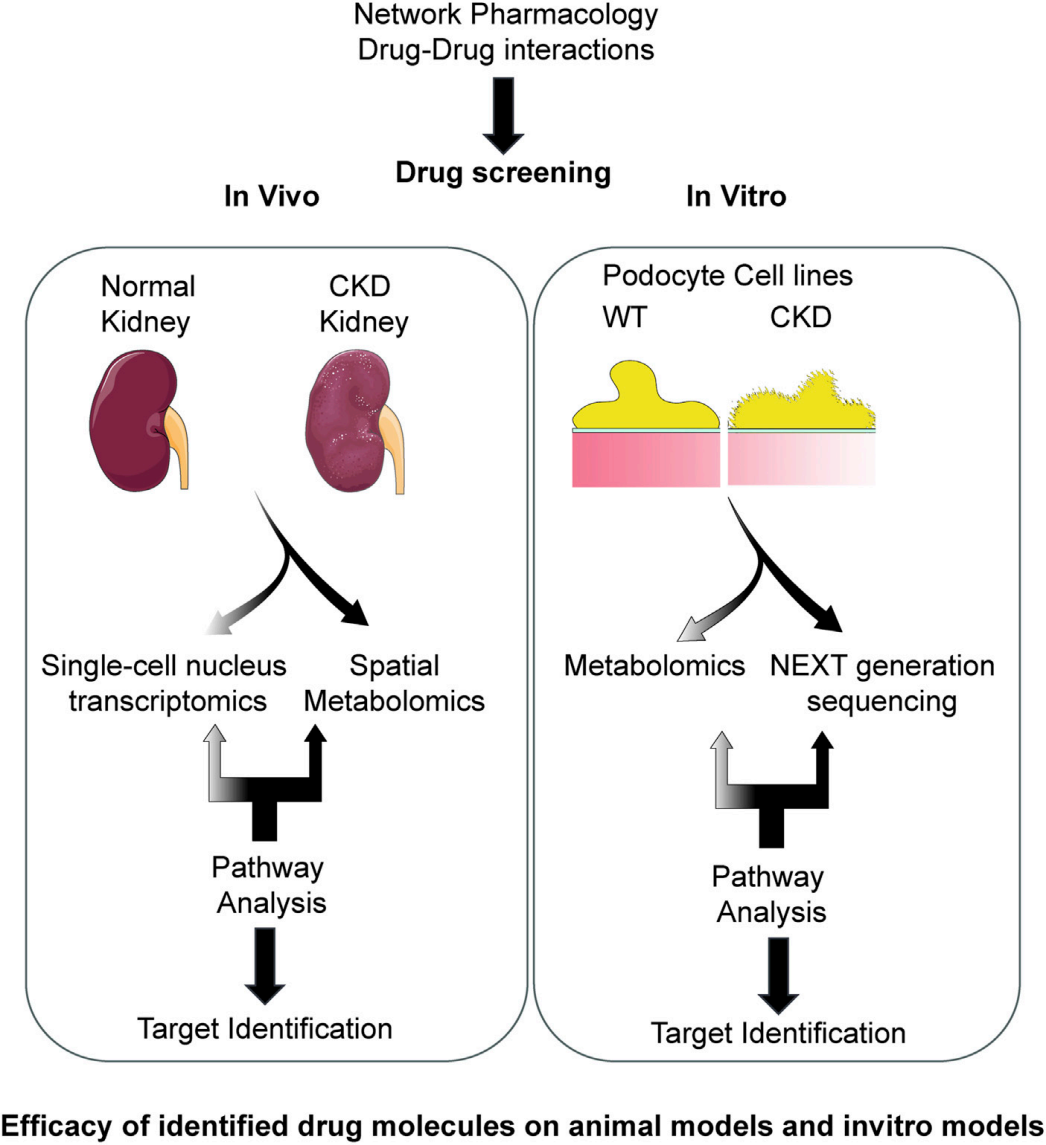
A schematic diagram showing the use of network pharmacology, drug-drug interactions in *in vitro* and *in vivo* screening. Components of this figure were created using Servier Medical Art templates, which are licensed under a Creative Commons Attribution 3.0 Unported License; https://smart.servier.com.

The one-drug/one-target/one-disease approach to drug discovery is presently facing many challenges of safety, efficacy, and sustainability. Today, network pharmacology and molecular docking are the most utilized system biology approaches and are used in drug discovery to study the mechanism of action of small-molecule entities or natural products in the management of CKD. These techniques help us identify therapeutic targets, understand drug-drug interactions, and predict the interventions on the complex signaling pathways related to CKD progression. Combining network pharmacology and molecular docking provides a comprehensive approach to identifying potential therapeutic options and offers a promising approach for identifying effective biomarkers for CKD. Integrated analyses of data from various sources, such as next-generation sequencing analysis, single-cell transcriptomic and single-cell genomics analysis, and microarray and metabolomics data, can provide a comprehensive overview of the molecular changes associated with CKD. Metabolomics can be utilized to screen differentially expressed metabolites in kidney tissues and to predict potential targets using relevant databases. The interaction networks among endogenous metabolites and target proteins have been established by integrating differentially expressed metabolites and proteins associated with CKD identified through proteomics. Bioinformatics analyses such as gene ontology (GO) analysis, Kyoto encyclopedia of genes and genomes (KEGG) pathway analysis, and weighted gene co-expression network analysis (WGCNA) can identify key signaling pathways and potential biomarkers associated with CKD development and progression. Pharmacological approaches can provide additional insights through the analysis of drug–gene interactions and the identification of potential therapeutic targets for CKD. The biological functions of the candidate metabolites and their effects on downstream pathways can be verified. Figure 1 demonstrates the schematic chart for the target identification and drug screening against CKD.

This article Research Topic aims to 1) identify effective biomarkers for the diagnosis and treatment of CKD by integrating bioinformatics and pharmacological approaches; 2) conduct bioinformatics analyses, including GO analysis, KEGG pathway analysis, and WGCNA, to identify key signaling pathways and potential biomarkers; 3) explore potential pharmacological implications by analyzing drug-gene interactions and identify potential therapeutic targets; and 4) examine immune infiltration features and related hub genes that might play a role in CKD development.

In this Research Topic, Patil et al. elucidate the disulfiram-modulated targets and pathways in renal fibrosis. The authors have analyzed the protein–protein interactions, pathway enrichment, and cluster and gene ontology between disulfiram and renal fibrosis by using the molecular docking procedure. Moreover, molecular dynamics simulation was performed to infer protein–ligand stability, and conformational changes were analyzed by free energy landscape. This analysis determines the renal protective activity of disulfiram and paves the way for experimental investigation to repurpose disulfiram for treating renal fibrosis. Another manuscript by Huang et al. investigates the correlation between hemoglobin A1c (HbA1c) levels and all-cause and cardiovascular disease mortality in elderly individuals with non-diabetic CKD.

In conclusion, a better understanding of these interactions can inform the development of targeted therapies that can improve the diagnosis and management of CKD.
